# Exploring the Efficacy of the Occlusal Stamp Technique: A Dual Perspective

**DOI:** 10.7759/cureus.70672

**Published:** 2024-10-02

**Authors:** Vaibhav Pipare, Soniya B, Joyeeta Mahapatra, Anuja Ikhar, Komal Agrawal

**Affiliations:** 1 Conservative Dentistry and Endodontics, Sharad Pawar Dental College and Hospital, Datta Meghe Institute of Higher Education and Research, Wardha, IND

**Keywords:** composite restoration, minimally invasive dentistry, minimally invasive restoration, occlusal stamp technique, replicating occlusal anatomy

## Abstract

Objectives of contemporary restorative dentistry include achieving proper tooth structure and minimizing the duration in the dental chair. There is a chance of contamination between the layers when posterior teeth are progressively restored with composite resin. The occlusal stamp technique is a brand-new, cutting-edge way to restore mostly Class I and occasionally Class II restorations with nearly ideal occlusal topography. It was created to make dental professionals' jobs easier and produce both practical and aesthetically pleasing outcomes. This case study demonstrates how the stamp technique was applied successfully to a simple Class 1 composite restoration. By duplicating the initial, basic tooth structure, the goal was to obtain precise anatomy in a matter of minutes by simulating occlusal anatomy.

## Introduction

Biomimetic restoration refers to the process of restoring a tooth to its original anatomical configuration [1]. Due to the increasing demand for aesthetic treatment, contemporary dental practice utilizes resin composite whenever a restoration for a tooth is needed. With the advent of minimally invasive dentistry (MID), a sharp rise is seen in the use of adhesive materials for the restoration of the posterior teeth [2]. Nowadays, silver amalgam is rarely the choice of restorative material due to its unappealing look and the health risks associated with mercury [3].

Achieving the cusp-fossa conjunction of teeth in a direct composite restoration is challenging, and achieving the occlusal harmony requires both the operator's competence and valuable clinical time. Compared to silver amalgam restoration, composite restorations take additional time for final polishing and occlusal corrections. Dr. Waseem Riaz developed the occlusal stamp technique to replicate tooth form and occlusion precisely [4].

An innovative approach to accurately restoring Class I and Class II restorations on the occlusal surface is called the "stamp technique." It is specifically designed for teeth with optimum occlusal structure and undamaged marginal ridges, even when dental caries is evident. To replicate the original anatomy, this approach entails making an occlusal stamp, excising carious tissue, and pressing the stamp into the final composite increment before final curing [5].

## Case presentation

Case one

**Figure 1:**
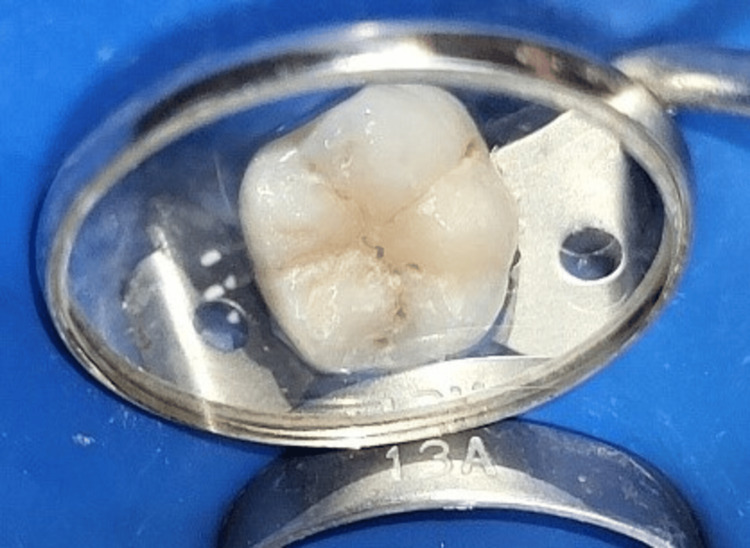
Preoperative clinical view of tooth number 36 showing pits and fissure caries

A 24-year-old male reported to the outpatient department with the complaint of a decayed tooth in the lower left back tooth region. The patient did not give a history of any discomfort or sensitivity related to the tooth. The patient gave no relevant medical history. Upon clinical examination, pits and fissure caries were identified with tooth number 36 without the involvement of the marginal ridge (Figure 1). As a result, it was decided to restore the tooth using composite material with the stamp technique.

**Figure 2:**
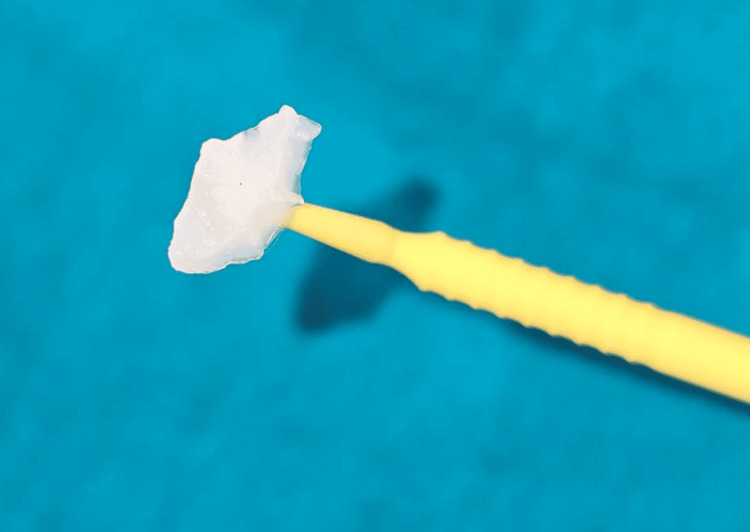
Composite stamp obtained from the occlusal surface of tooth number 36

The tooth in concern was meticulously isolated using a rubber dam, ensuring precise and effective treatment. A thin layer of petroleum jelly was gently applied to the occlusal surface using a specialized applicator tip. This acted as a crucial separating agent, ensuring smooth and precise application. The microbrush was carefully trimmed at the tip with scissors to enhance its ease of handling. The flowable composite material (Tetric N-Flow, Ivoclar Vivadent, Inc., Amherst, NY) was carefully applied to the occlusal surface, covering the required occlusal anatomy. The microbrush was then gently placed over the composite material and light-cured. Finally, an occlusal stamp was created, accurately making a negative replica of the occlusal surface of tooth number 36 (Figure 2).

**Figure 3:**
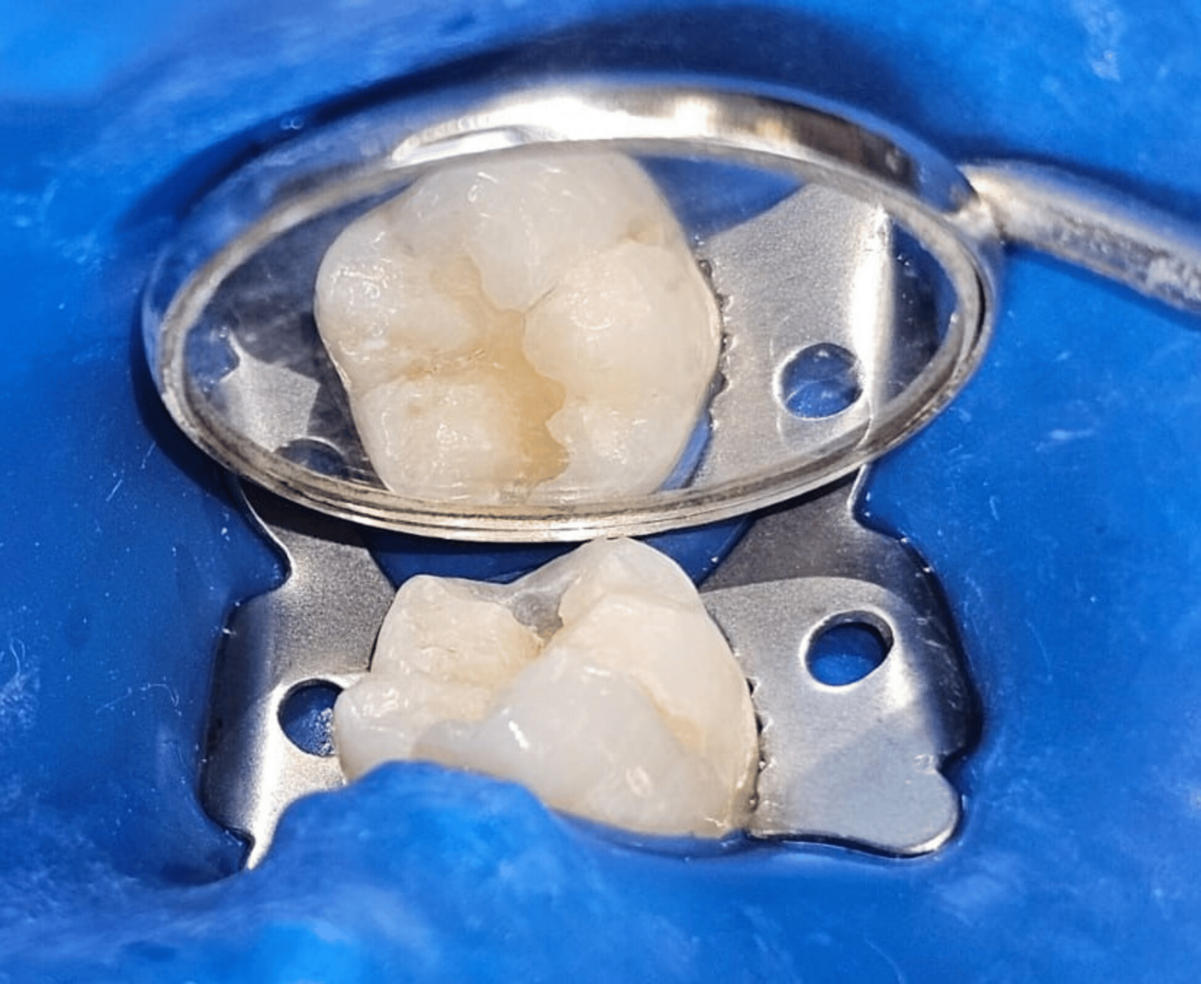
Caries excavation done on tooth number 36

The caries excavation was limited to the margins of the decayed area. This area was removed using an air-rotor and a large round bur at high speed (Figure 3). Thirty-seven percent of orthophosphoric acid (Ivoclar Vivadent, Inc.) was used for etching for 30 seconds, after which the material was rinsed off under running water for 10-20 seconds. After rinsing, the tooth was dried. The universal bonding agent (3M ESPE Single Bond Universal Adhesive, 3M, Maplewood, MI) was carefully applied and then light-cured for 20 seconds.

**Figure 4:**
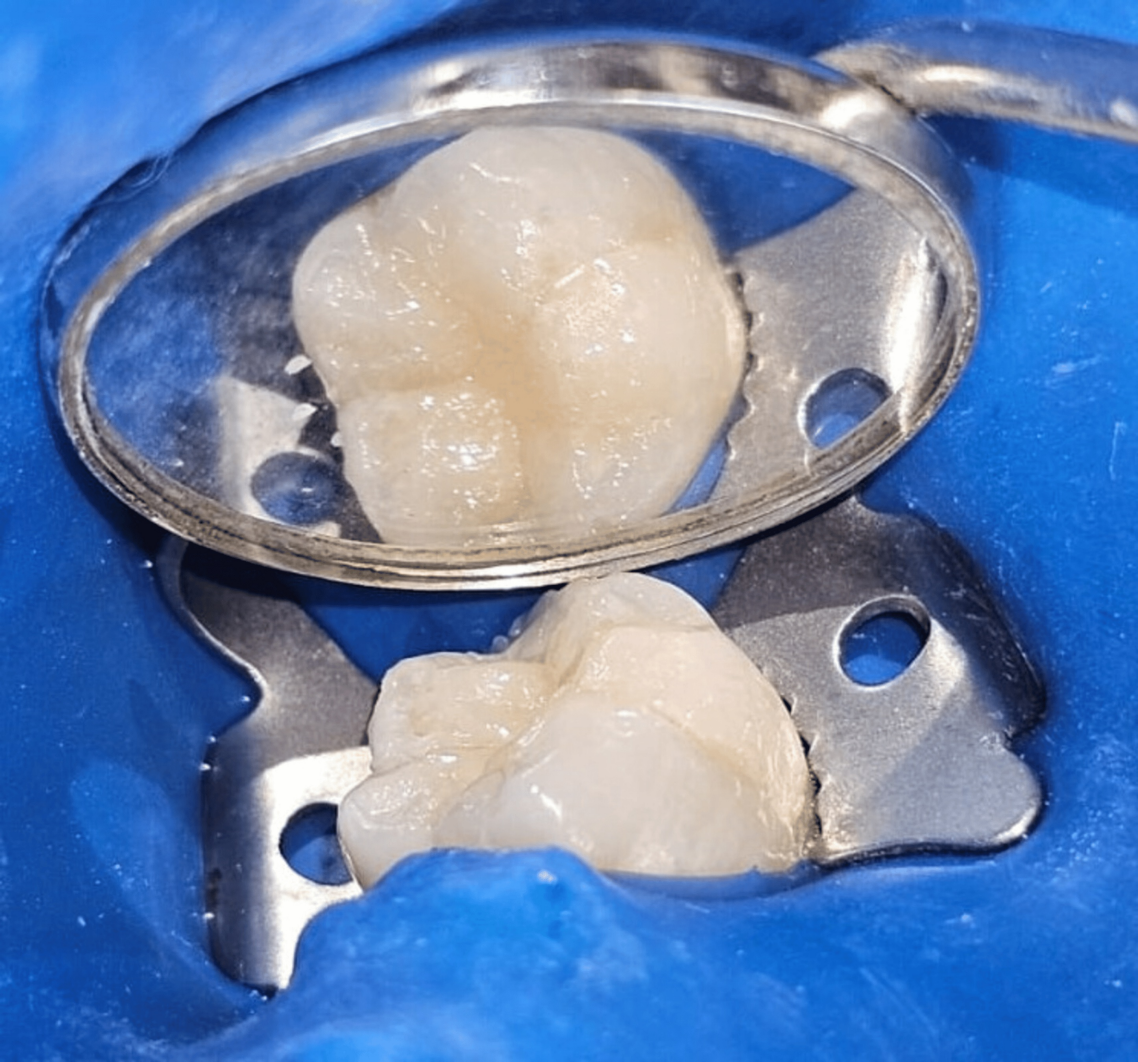
Postoperative clinical view of tooth number 36

The cavity was incrementally filled with resin composite material (TPH Spectra ST composite, Dentsply Sirona, Charlotte, NC). The filling was done up to 1 mm below the occlusal surface. After the filling, it was then light-cured for 30 seconds to harden and secure the composite material in place. The application of the final composite layer was followed by the careful placement of cling film on the occlusal surface before curing. To replicate the original anatomy, the composite stamp of the occlusal surface taken before the beginning of the procedure was used to press on the composite and cure it for 30 seconds. Following the completion of the restoration's final cleaning and finishing, the rubber dam was taken out (Figure 4).

Case two

A 27-year-old female patient, with no significant medical history and a non-contributory dental history, presented to our dental clinic with discomfort localized to the lower right posterior region. The patient reported intermittent episodes of sensitivity to hot and cold stimuli in the affected area, particularly when consuming food or beverages. On examination, the patient exhibited good oral hygiene practices and showed no signs of systemic illness.

Clinical evaluation revealed a Class I carious lesion in tooth 48, which was identified during an occlusal and proximal surface examination. The lesion presented as a distinct darkening within the occlusal pit, accompanied by subtle discoloration along the proximal surface. Palpation of the affected tooth elicited mild tenderness, consistent with the patient's reported discomfort. There were no signs of swelling, abscess formation, or gingival inflammation in the surrounding periodontal tissues.

**Figure 5:**
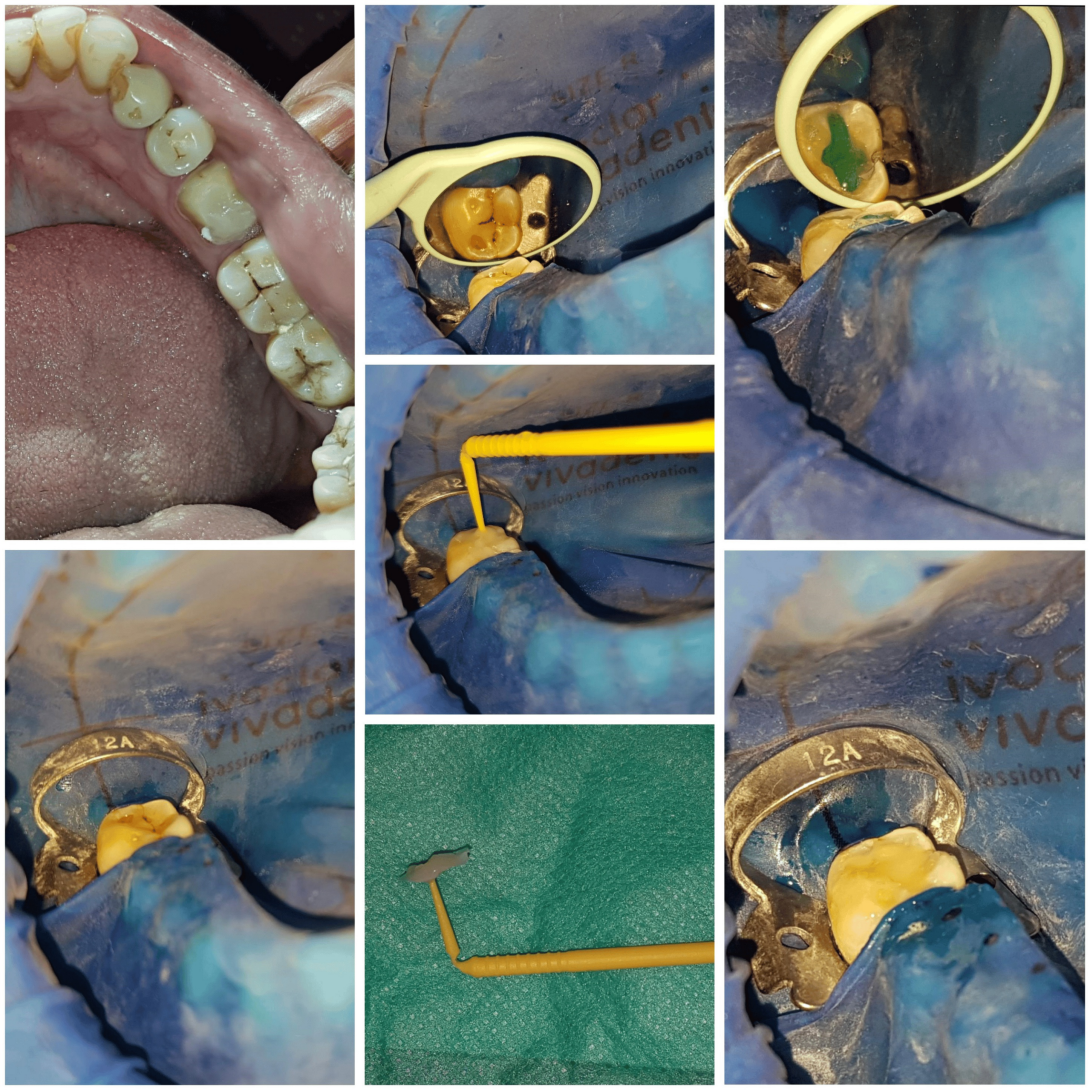
Step-by-step occlusal stamp technique

Radiographic assessment, utilizing occlusal, periapical, and bitewing views, confirmed the presence of radiolucency extending into the dentin, indicative of a Class I carious lesion. Despite the radiographic evidence of caries, there were no discernible signs of pulp involvement or periapical pathology. The adjacent teeth appeared sound, with mild evidence of carious lesions or restorative interventions (Figure 5).

Given the patient's desire for a conservative treatment approach that prioritized the preservation of healthy tooth structure, the decision was made to utilize the occlusal selective caries removal (OCTRADAM) technique. This approach involves removing only the carious tissue from the occlusal surface, leaving the surrounding sound tooth structure intact. Informed consent was obtained after detailed information about the planned procedure, including its steps, expected outcomes, and possible complications.

Following the patient's consent and adequate local anesthesia administration, isolation of the operative field was achieved using a rubber dam to ensure optimal moisture control and patient comfort. The OCTRADAM technique was employed to address the Class I carious lesion in tooth 48 conservatively.

## Discussion

Restoring a posterior tooth anatomy can be challenging using direct composite resin restorative material. For dental professionals, reconstructing the posterior teeth's occlusal surface morphology can be difficult. Dental professionals confront several difficulties while reconstructing the posterior tooth occlusal surface. Replicating posterior teeth precisely is necessary to maintain function and appearance because of their complex architecture, including pits and grooves. Establishing proper occlusal contact is important because it has to be aligned with the teeth on each side, which can be challenging due to wear patterns and changes over time. Achieving the ideal balance between strength, durability, and aesthetics when choosing a restorative material can also be challenging. Strong bonding between the material and the tooth structure is another issue, particularly when there is significant decay or damaged enamel. Collaboration with the patient is crucial since some people may find it difficult to keep their mouths open or may experience anxiety when having surgery. Efficient bonding depends on controlling moisture, and tongue and saliva movement might make it difficult to keep the area dry. Elaborate instances may become even more complex due to time restrictions in busy practice environments. To improve patient satisfaction, dentists also need to help patients with postoperative discomfort and make sure the rebuilt surface can endure the pressures of mastication over time. Lastly, a natural look is still desirable even with posterior restorations, especially in more noticeable places. Skill, expertise, and good patient communication are all needed to navigate these challenges.

The new stamp method overcomes several issues with traditional methods and provides a creative approach to applying composite restorations. This approach aims to address the specific problems and provide improved treatment outcomes. For optimal treatment results and overall patient satisfaction, pit and fissure caries must be maintained structurally [6]. Appropriate finishing and polishing dental restorations are essential to enhance their durability and visual appearance. The occlusal index increases surface hardness by protecting the resin composite from atmospheric oxygen, ensuring proper polymerization [7,8]. It has been shown that improving the appearance and durability of dental restorations requires careful finishing and polishing techniques. It's also critical to recognize that the impression materials are expensive. However, it is necessary to investigate more affordable options of materials for making stamps, such as polymethyl methacrylate, pattern resin, bite registration material, vacuum-formed templates, pit and fissure sealants, and gingival dam material. Enhancing the surface's smoothness is crucial, and this may be accomplished by pre-copying the occlusal surface.

Resolving occlusal issues resulting from modifications in occlusal morphology is crucial [9]. Primary occlusal trauma can be brought on by interference or early contact resulting from inadequate occlusal adjustments. Because the natural anatomy of the tooth is preserved, this approach has a considerable benefit [10]. It prevents dental drilling-induced microfractures from occurring, ensures the continuity of the filling's surface and borders, and cuts down on the time required for occlusal correction [11]. Although it works well for Class II cavities as well, the stamp approach is mostly employed for Class I cavities. But before using this method, it's imperative to carry out a comprehensive assessment.

## Conclusions

Both stamp restoration techniques and conventional procedures have their advantages and disadvantages. One of the most notable features of the stamp technique is its ability to produce occlusal anatomy with precise anatomical shapes or biomimicry without requiring operator knowledge. In addition, its occlusal topography provides a far greater level of accuracy and precision than the manual method. This novel approach shortens working time while also simplifying composite polishing. Conversely, because of the increased shrinkage of the composite during polymerization, the traditional approach takes longer and needs more experience, but it is appropriate for bigger, irregularly shaped caries.
